# Perceived communication effectiveness in implementation strategies: a measurement scale

**DOI:** 10.1186/s43058-022-00284-4

**Published:** 2022-04-08

**Authors:** Xiaoquan Zhao, Heather Toronjo, Cameron C. Shaw, Amy Murphy, Faye S. Taxman

**Affiliations:** 1 Department of Communication, George Mason University, 3D6, 4400 University Drive, Fairfax, VA 22030 USA; 2 Center for Advancing Correctional Excellence, Schar School of Policy and Government, George Mason University, Arlington, USA

**Keywords:** Communication, Implementation strategies, Perceived effectiveness, Measurement

## Abstract

**Background:**

Communication-based activities and products (i.e., training programs, webinars) are a critical component of implementation strategies that relay information to various audiences. Audience perceptions of communication effectiveness contribute important insight into the processes and mechanisms through which an implementation effort may succeed or fail. To advance research on this front, a psychometrically sound instrument for measuring perceived communication effectiveness (PCE) is needed.

**Methods:**

An expert panel identified the theoretical foundations and conceptual domains of PCE and drafted preliminary items. Five focus groups of correctional professionals who had recently completed an implementation leadership training reviewed the items and provided feedback for refinement. Revised items were then included in a survey-based evaluation of an ongoing eLearning curriculum designed to improve the practices used by front-line probation officers in supervising individuals in the field. The factorial structure of a final 6-item scale as well as its convergent, divergent, and predictive validity was evaluated using data from the evaluation surveys (*N*_follow-up_ = 358, *N*_baseline+follow-up_ = 159).

**Results:**

Confirmatory factor analysis of the final scale of PCE demonstrated adequate fit. PCE was strongly correlated with measures of implementation outcomes (acceptability, *r* = .819, *p* < .001; appropriateness, *r* = .809, *p* < .001; and feasibility, *r* = .754, *p* < .001), yet uncorrelated with a scale of need to evaluate (*r* = − .051, *p* = .422), demonstrating both convergent and divergent validities. The predictive validity of PCE was evidenced by significant associations between PCE and key training outcomes, including perceived staff use of evidence-based practices (*β* = .230, *p* < .05), agency climate (*β* = .261, *p* < .05), and value concordance (*β* = .209, *p* < .05), after controlling for baseline values and other confounders.

**Conclusions:**

The PCE scale is psychometrically sound and can be a useful tool for gauging audience receptivity to and the potential impact of communication-based implementation activities and products.

**Supplementary Information:**

The online version contains supplementary material available at 10.1186/s43058-022-00284-4.

Contributions to the literature

- Communication is an integral part of implementation processes. Yet, no measurement tool currently exists to assess the audience perceptions of the effectiveness of communication activities and products in implementation efforts.
- A brief scale for measuring perceived communication effectiveness (PCE) was developed with evidence of sound psychometric properties.
- The scale can be a valuable tool for gauging audience reactions to dissemination and diffusion efforts and their potential impact on practice.

## Background

Implementation strategies are designed to facilitate the adoption and adaption of innovations or reforms in an organization. Strategies include dissemination, quality improvement, implementation processes, integration with existing processes, capacity-building, and scale-up with varying impact at the individual and organizational levels. Most implementation strategies include a communication component, wherein the new evidence-based practice is introduced to and promoted among organizational leadership, staff, and other stakeholders through training, workshops, webinars, brochures, videos, etc. An important question is whether those participating in planned change strategies perceive both the innovation and the implementation efforts to be effective. For example, most new innovations involve some type of training, but studies find that audiences tend to recall about 10% of the presented material [1], with few efforts resulting in implementation. As indicated by meta-analyses, trainings that do not focus on procedural or actionable strategic knowledge are less likely to be perceived as feasible in the workplace [2, 3]. Most trainings tend to focus more on disseminating an array of information rather than building knowledge about how to use the material in routine everyday decisions. Without consideration for the effectiveness of implementation efforts, the prospect remains uncertain how well innovation will improve operations.

Advancing the utility of implementation strategies requires a better appreciation of whether these strategies resonate with those who are exposed to them. Unresolved questions that Proctor and colleagues [4] outlined earlier still stand in the field, including (1) assessing the effectiveness of implementation strategies on key outcomes such as acceptability, appropriateness, feasibility, and fidelity to evidence-based practices (EBPs), especially those that are designed to impart knowledge, skills, and attitude changes, and (2) designing and evaluating strategies that communicate vital information that can be used in everyday work practices. These two questions intertwine the disciplines of implementation and communication sciences which collectively serve to build knowledge about the uptake of revised practices and procedures. Implementation and communication sciences offer different but complementary perspectives on the meaning of effectiveness. Together, these two disciplines provide a useful platform for assessing whether exposure to dissemination strategies can bring about change in implementation.

EBPs or information on research-based practices found to improve outcomes can identify *what* should be implemented based on efficacy and effectiveness studies, whereas implementation science can identify the *process* by which routine practices can be altered and improved [5]. Communication science plays an integral role in ascertaining whether implementation processes provide critical information to design, refine, and structure new procedures. Communication “focuses on how people use messages to generate meanings within and across various contexts and is the discipline that studies all forms, modes, media, and consequences of communication through humanistic, social scientific, and esthetic inquiry” [6]. A recent systematic review of 27 implementation theories and models found that communication was an explicit concept in 16 models, and the remaining 11 models acknowledged the role and importance of communication implicitly [7]. Despite the recognition of communication’s importance, formal attention to communication-related issues is still largely lacking in studies of effective implementation strategies.

Communication-based activities and products (e.g., training programs, webinars) are a critical component of implementation strategies that relay information to various audiences. Attention to audience perceptions of communication effectiveness (perceived communication effectiveness, hereafter PCE) is aligned with potential implementation outcomes such as acceptance, appropriateness, feasibility, and fidelity. An emphasis on PCE contributes important insight into the processes and mechanisms through which an implementation program may succeed or fail. In the same vein, PCE has critical diagnostic value for examining the quality of communication that affects the success or failure of implementation activities.

To advance research on PCE in implementation science, a carefully crafted and validated measurement tool is needed. Several comprehensive reviews of measurement instruments in the implementation literature note that many existing measures lack conceptual grounding and systematic testing [8, 9]. In this article, we present a PCE scale that is theoretically grounded; incorporates research in communication, psychology, and implementation science; and has gone through a rigorous development and validation process. The initial testing of the PCE scale was conducted in the context of EBP promotion through in-person and online staff training for individuals who work in corrections or probation agencies. Constructed intentionally for broad use, this scale should prove useful in other implementation research and application contexts.

## Perceived effectiveness

The development of the PCE scale draws heavily from prior work on perceived message effectiveness (PME) in the persuasion and communication campaign literature. PME is “an estimate of the degree to which a persuasive message will be favorably evaluated—in terms of its persuasive potential—by recipients of that message” [10]. PME has been widely used in communication interventions, both as a tool to assess message potential in formative research [10, 11] and as a surveillance device to monitor audience receptivity during campaign implementation [12, 13].

As an example, a PME measure called perceived argument strength has enjoyed relatively wide adoption, particularly in tobacco and drug prevention research [14]. Developed as a complement and potential alternative to the traditional thought-listing technique that persuasion researchers have often used to assess argument/message strength [15], the perceived argument strength scale is a relatively brief instrument that can be administered easily as part of an outcome questionnaire following message exposure. Testing of the perceived argument strength scale has shown sound psychometric properties, including strong convergence with the traditional thought-listing method [14]. This scale has been used in a variety of message testing studies, typically in health contexts, that assess message/argument potential to motivate attitude and behavior changes [16, 17].

Existing measures of PME tend to focus heavily on relatively brief persuasive messages in broad-reaching media, such as television/video advertisements or warnings on product packaging [18, 19]. For example, PME has been a central research tool for developing and evaluating youth tobacco education campaigns sponsored by the US Food and Drug Administration (FDA) [12, 20, 21]. In these campaigns, the assessment of PME has almost exclusively focused on brief, simple, disparate educational messages (e.g., a 30-s PSA on dangerous chemicals in cigarette smoke). For the complex messaging required in implementation efforts, a measurement tool is needed that is broader in scope and more responsive to the unique characteristics of associated communication activities and products. To reflect this broader scope of application, we term the new scale “perceived communication effectiveness” and anticipate its applicability to diverse types of communication including, but not limited to, brief persuasive messages.

The growing interest in the utility of PME in communication campaigns has led to a closer examination of the concept and its measurement in recent years, highlighting important gaps in the literature [22–25]. Issues that stand out include a lack of conceptual clarity and theoretical development [25, 26], unprincipled heterogeneity in the operationalization of the concept [22, 23], and the quality of the evidence base for PME’s utility, particularly as a tool for message pretesting [23, 24]. In general, these concerns echo the need for both careful conceptualization and systematic examination of psychometric properties of new scales in the implementation literature [8, 9]. Development of the current PCE scale was mindful of these issues and prioritized rigor in both conceptualization and empirical testing.

## Theoretical grounding

The PCE scale is grounded in relevant theories and research in communication, psychology, and implementation science. The selection of theories to guide the current work balances considerations on multiple fronts: the ability to illuminate key constructs and processes in PCE, application in relevant previous research, and the need for a contextualized perspective on effectiveness in implementation strategies. The primary guiding framework for the conceptualization of PCE is William McGuire’s communication and persuasion model [27], a theory that has broad influence on the persuasion and campaign literature [28] and previous PME work [22, 29]. According to this model, communication and persuasion occur through a series of attentional, cognitive, and decision-making steps. For a message to be persuasive, it must first be received and then generate yielding. The reception stage includes exposure, attention, interest, comprehension, and knowledge acquisition. The yielding stage includes attitude change, memory, retrieval, decision-making, action, reinforcement, and consolidation. Perceived effectiveness is typically focused on the initial steps in the persuasion process (i.e., the reception stage; for a different point of view, see [19, 30]) because strong performance in reception lays the foundation for attitude and behavior change. The latter outcomes, while more directly relevant to the objectives of many communication interventions and implementation activities, are typically difficult to detect without substantial time and investment. For formative research and intermediate evaluation, variables from the reception stage can serve as useful indicators of the potential impact of communication messages and products in the yielding stage.

Another theory that informs PCE scale development is the elaboration likelihood model (ELM) [31], a theory about information processing that guides some of the previous PME work [14]. ELM posits that human cognition draws on a limited pool of cognitive resources. As a result, not all information is processed deeply and thoroughly. Determinants of message elaboration are typically factors related to people’s motivation and ability to process. On the motivation front, a key variable is issue relevance. People are generally more likely to pay attention to information that matters to them. If they do not consider incoming information to be personally relevant, audiences are not likely to engage with the message on a deep level. In terms of processing ability, people’s prior knowledge and the amount of distraction they have to endure in a cluttered informational environment are key factors that can directly affect comprehension of communication products. Converging with McGuire’s model in emphasizing interest and comprehension in communication reception, ELM considers engagement and issue importance to be critical antecedents to eventual communication impact.

The development of the PCE scale is also informed by implementation science literature, which focuses on understanding how different implementation strategies affect innovation adoption and adherence. Implementation strategies range from group learning and trainings to quality improvement processes [32]; these strategies are designed to propel various outcomes such as the belief that core features of the innovation are understood, that the innovation is appropriate for the setting and/or population, that the innovation is accepted by staff or administrators, and that the innovation can be implemented. Since implementation strategies are designed to facilitate actionable use of knowledge, an emphasis on perceived effectiveness will inform whether the strategy succeeded in persuading the audience that the innovation has utility. That is, perceived effectiveness can help assess the degree to which the implementation strategy achieved the outcome of a viable idea, enhanced or broadened perspectives on current practice, and/or served as a mediator or moderator for other implementation outcomes.

## Conceptual domains of the PCE scale

**Fig. 1:**
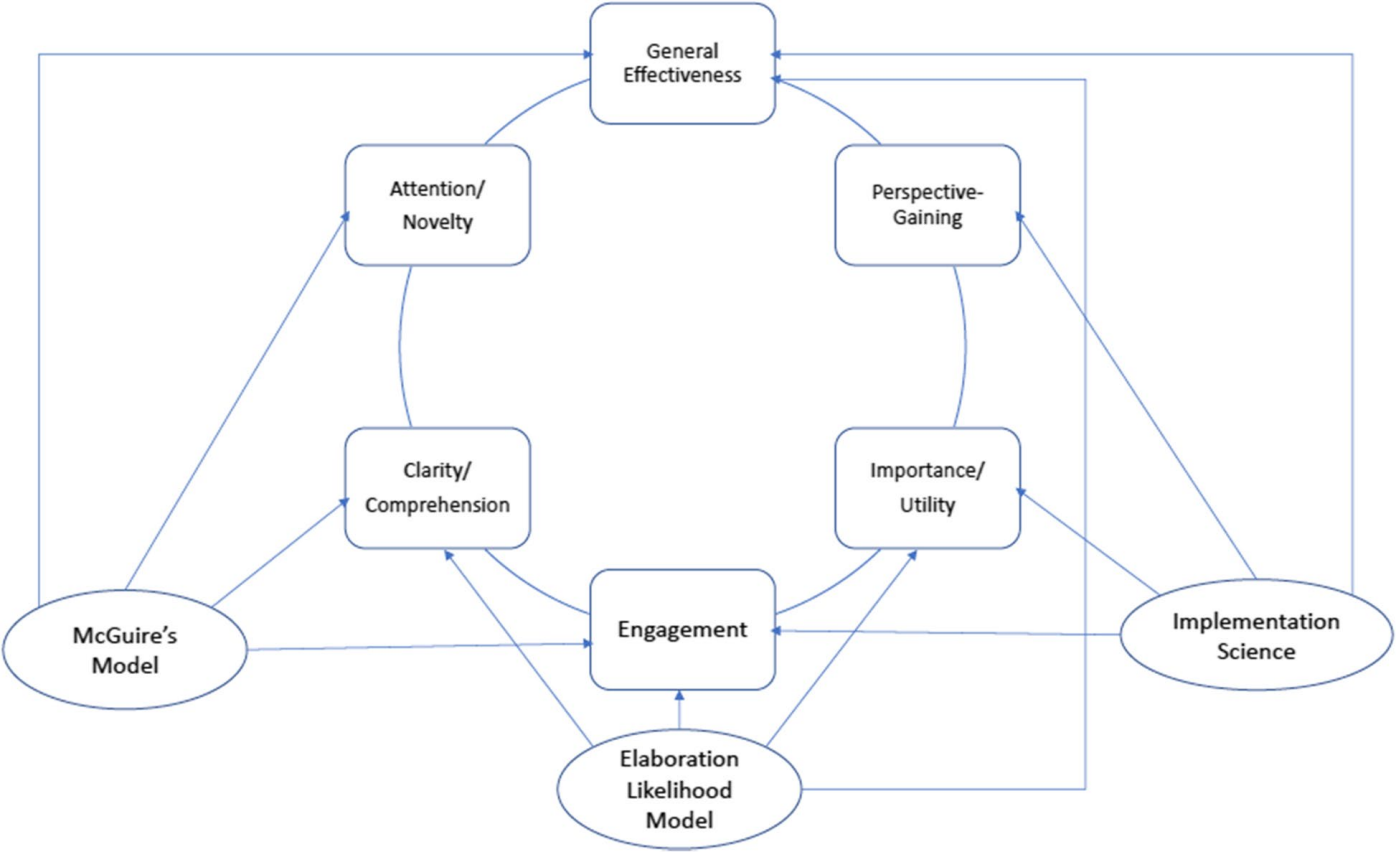
Conceptual dimensions in PCE and the underlying theories/literature

Based on relevant theory and research, we identified the following dimensions of the PCE scale: attention/novelty, clarity/comprehension, engagement, importance/utility, perspective-gaining, and general effectiveness. The selection of these dimensions focused on the reception stage in McGuire’s model while also integrating key insights from ELM and current research on implementation processes and outcomes. Together, these dimensions represent a broadened perspective on communication effectiveness in the implementation context, covering both important constructs that have been widely engaged in previous PME work and additional indicators that reflect the distinctive nature of implementation communication. Some scale dimensions highlight points of convergence between different theories and research traditions (e.g., comprehension and engagement are central to both McGuire’s model and ELM). Others capture unique considerations from a particular perspective (e.g., the importance of perspective gaining in implementation science). The conceptual linkages between the PCE dimensions and the underlying theories and literature are diagramed in Fig. 1.

The PCE scale is not meant to be a comprehensive assessment tool of communication effectiveness. The actual effectiveness of communication products and activities, much like that of entire implementation programs, is best assessed by examining the population and/or organizational impact over time. PCE is intended as a proximal outcome, serving primarily surveillance and diagnostic functions. For that reason, the PCE scale does not address program outcomes directly, nor does it intend to capture all processes and mechanisms contributing to eventual program success. The scale instead focuses on the most essential reactions of stakeholders and other audiences toward an implementation communication effort, with the theory-informed assumption that these reactions will reliably predict the actual impact of the communication effort. Finally, the development and validation of the PCE scale place a strong emphasis on usability, with the understanding that shorter, more compact scales are more likely to be adopted across research and application purposes [8].

## Study overview

In this article, we describe the development and validation of a brief PCE scale. Evidence will be reported on the scale’s factorial structure, convergent validity, divergent validity, and predictive validity. For convergent validity, we focus on the relationship between PCE and published measures of implementation outcomes. For divergent validity, we examine the association between PCE and an individual difference variable called need to evaluate, i.e., the chronic tendency to engage in evaluative responding [33]. The expectation is that PCE ratings will be independent of individuals’ tendency toward forming strong opinions. For predictive validity, we focus on PCE’s ability to predict specific learning outcomes of an ongoing training program.

## Methods

The scale development process consisted of four phases. In phase 1, the researchers convened an expert panel over multiple sessions to propose and discuss potential items. In phase 2, five focus groups of criminal justice professionals, who had recently completed a correctional training program on implementation, reviewed the preliminary items. The pool was refined based on participant feedback. Phases 3 and 4 occurred in the context of an ongoing training on improving the use of EBPs with community supervision professionals; phases 3 and 4 involved a different sample than phase 2. Participants in the training completed both a baseline and a post-training follow-up survey; the preliminary items for the PCE scale were included in the latter survey. In phase 3, we used data from the follow-up survey to examine the item properties and the factorial structure of two forms of the scale, eventually leading to a final scale of 6 items. In phase 4, the convergent, divergent, and predictive validities of the final PCE scale were assessed using data from both the baseline and the follow-up surveys. This study reports data following the STROBE checklist for observational studies.

### Phase 1—Construction of item pool

A group of experts from health communication (*N* = 2), implementation science (*N* = 3), justice and health advocacy organizations (*N* = 2), and criminal justice leadership (*N* = 1) met several times to discuss the potential conceptual coverage of PCE and to propose preliminary items. These experts were identified through existing networks of collaboration, including a large research collaborative called Justice Community Opoid Innovation Network (JCOIN) (www.jcoinctc.org). They brought together substantial expertise spanning across media campaigns, message design and testing, healthcare for justice populations, implementation research and practice, public policy advocacy, and organizational EBP adoption. The theoretical models and relevant research presented above were reviewed and deemed appropriate to serve as the conceptual foundation for the new scale. Key dimensions of the construct were identified through discussion, and consensus was reached on the final scale structure. The panel members then proposed and discussed the preliminary items. The process was iterated through several meetings and resulted in a pool of 34 items, with three to six items representing each of the six dimensions under the general construct of PCE. All items were crafted in such a way that they could be easily adapted for different communication products or activities, such as trainings, webinars, or issue briefs. General guidelines in the methodological literature on item writing were followed to ensure that each item was clear, concise, unambiguous, and as concrete as possible [34, 35]. All items were declarative statements suitable for Likert scale responses (strongly agree to strongly disagree).

### Phase 2—Focus group testing

Preliminary scale items were subjected to cognitive testing to verify content validity, clarity, and appropriateness for the scale’s intended purposes. A convenience sample of participants (*N* = 15) was recruited via a midwestern state Department of Corrections (DOC) with approval from the director. The focus group protocol was approved by the authors’ university’s institutional review board and available in Additional file 1: Supplemental Appendix A. Five focus groups were conducted between April and May of 2020. DOC employees had completed an in-person implementation leadership academy, a training designed to advance implementation efforts, between November 2019 and January 2020; they were subsequently invited to participate in 1-h focus groups about the experience. Due to COVID-19 pandemic restrictions, all focus groups were conducted virtually. Focus group participants included nine men and five women, with one person preferring not to disclose. Twelve of the participants were White, one was Black or African American, one was Hispanic or Latinx, and one preferred not to disclose. The focus groups started with open questions about experience with the training, including the overall evaluation and specific likes and dislikes. Participants then reviewed all 34 preliminary scale items and were probed for their thoughts and reactions regarding item clarity and applicability. The probing was structured in such a way that it touched on both the content and specific phrasing of the items. All focus groups were recorded. Transcripts of the focus groups and the research team’s field notes were analyzed through iterative reading and discussion to extract both general and item-specific feedback. Overall, participants felt the items were straightforward and appropriately reflected their training experience. One suggestion was to shorten the instrument to ensure attentive response and to accommodate variations in duration and style of training experiences. Other suggestions centered around modified wording to make items clearer and more neutral. Revised preliminary items based on feedback from the focus groups are presented in Additional file 1: Table S1.

### Phases 3 and 4—SUSTAIN evaluation

#### Background on training

The final phases of scale development and validation were conducted as part of the evaluation of an eLearning curriculum called SUSTAIN (Staff Undertaking Skills to Advance Innovation), which is designed to improve the practices used by front-line probation officers in supervising individuals in the field [36]. SUSTAIN aims to improve outcomes related to knowledge and skills regarding evidence-based practices developed for supervision agencies [37–39], supervisor coaching skills, and organizational learning climate and culture [37, 40]. Each SUSTAIN module has three levels of material (basic, intermediate, and advanced) for each of the six topic areas: risk-need-responsivity (RNR), motivation and engagement, case planning, problem solving, desistance, and criminal lifestyle.

#### Study design

The evaluation of SUSTAIN consisted of a baseline survey and a post-training follow-up survey. Starting in February 2019, SUSTAIN was rolled out over several months in 15 county-level probation agencies in a southwestern state. Following the baseline survey, the agency staff received access to SUSTAIN and were instructed to complete the modules over a 6-month period. The entire SUSTAIN curriculum requires about 20 h to complete. In August 2020, the research team initiated the follow-up survey of 571 agency staff who completed SUSTAIN, including those who had not completed the baseline survey. The response rate for the follow-up survey was 62.7% (*N* = 358). Of the 358 respondents, 44.4% (*N* = 159) completed both the baseline and follow-up surveys. The SUSTAIN evaluation project was approved by the authors’ university’s institutional review board.

#### Variables

With the exception of PCE and a knowledge test, all key variables in the SUSTAIN evaluation surveys were published scales. The knowledge test (13 multiple-choice items) was expressly developed for SUSTAIN, assessing knowledge of EBPs including RNR principles, strengths-based supervision, case planning, and problem solving. Other survey measures included perceptions of the staff’s use of EBPs (4 items) [41, 42], the agency’s learning climate (16 items) [40, 43], and the degree to which the respondent was committed to the organization based on a set of shared values (i.e., value concordance, 8 items) [44], all of which were measured with 4-point Likert responses from 1 (strongly disagree) to 4 (strongly agree). Respondents were also asked about their perceptions of the acceptability (4 items), appropriateness (4 items), and feasibility (4 items) of the SUSTAIN training, all of which were reported with 5-point Likert responses from 1 (strongly disagree) to 5 (strongly agree) [45].

The variables mentioned above were included in both baseline and follow-up surveys. The follow-up survey also included the 34 preliminary PCE items with 7-point Likert responses from 1 (strongly disagree) to 7 (strongly agree), as well as a 16-item scale measuring individual need to evaluate [33] with five response options from 1 (extremely uncharacteristic) to 5 (very characteristic).

#### Analysis strategy

To examine the psychometric properties of the PCE scale, we conducted three types of analysis. We first performed confirmatory factor analyses (CFAs) to ascertain the factor structure of the scale. We used CFAs instead of exploratory factor analyses (EFAs) because of the strong conceptual foundation and clear a priori dimensionality of the scale [35, 46]. We estimated two forms of the scale, one with 21 items and another, which we focus on in this study, with 6 items. The CFAs were conducted using Mplus 8.0, and model fit was assessed using the following guidelines: comparative fit index (CFI) and Tucker-Lewis Index (TLI) at or above .95, root mean square error of approximation (RMSEA) at or below .06, and standardized root mean square residual (SRMR) at or below .08 [46, 47]. The chi-square test was not used as a primary criterion for model fit due to its known tendencies to overreject with large samples [47]. Internal consistency of the scales was evaluated upon evidence of adequate fit of the CFA models.

We then investigated the convergent and divergent validity of the final PCE scale by examining its correlations with training-specific evaluation measures of acceptability, appropriateness, and feasibility, plus the need to evaluate the scale. We expected PCE to correlate with the other evaluation measures but not with the need to evaluate.

Finally, we conducted a series of linear regressions to examine the PCE’s ability to predict knowledge of EBPs, perceptions of colleagues’ use of EBPs, agency climate, and value concordance, after controlling for baseline values as well as age, race/ethnicity, position, and county size. All races/ethnicities outside of White and Latinx were collapsed into a single category due to small sample sizes. Counties were collapsed into small, medium, or large based on population size. Significant coefficients for PCE were taken as evidence of the predictive validity of the scale. In all analyses, missing data were handled by listwise deletion.

## Results

### Sample

Sample characteristics for both the full follow-up sample (*n* = 358) and the matched longitudinal sample (*n* = 159) are reported in Table 1. For the full sample, the mean age was 41 years old. Half of the full sample (50.3%) identified as White, 18.7% as Latinx, and 14.2% as either American Indian, Asian, Black, multiethnic, or “others.” A majority (65.9%) were officers, 19.6% were supervisors and upper-level management, and 14.5% did not report their position. Nearly two-thirds (61.2%) represented two large agencies located in counties with a population greater than 1,000,000. Just over one-quarter (26.8%) were from mid-sized agencies with county populations between 100,000 and 1,000,000, and 12% were from small agencies with county populations below 100,000.

**Table 1:** Demographics of follow-up survey respondents (full sample) and matched baseline-follow-up respondents (longitudinal sample)

Characteristic	Full sample (*n* = 358)		Baseline/follow-up (longitudinal) sample (*n* = 159)	
	%	Mean (SD)	%	Mean (SD)
Age		41.61 (10.76)		41.78 (10.38)
Race/ethnicity*				
White	50.3		66.7	
Latinx	18.7		18.2	
Others	14.2		15.1	
Did not answer	16.8		0.0	
Position				
Officer	65.9		64.8	
Management	19.6		23.9	
Did not answer	14.5		11.3	
County size*				
Small (under 100K)	12.0		12.6	
Mid (101K to 1 million)	26.8		35.8	
Large (over 1 million)	61.2		51.6	

*p < .05 in comparison with the full and longitudinal samples

The mean age of the longitudinal sample was also 41 years old. A majority of the sample identified as White (66.7%), followed by Latinx (18.2%) and another race/ethnicity (15.1%). Most respondents were officers (64.8%), followed by management (including mid- and upper-level; 23.9%). The remaining 11.3% did not provide their position. Approximately half of the sample (51.6%) represented one large agency in a major metropolitan area. Respondents from mid-sized and small counties made up 35.8% and 12.6% of the longitudinal sample, respectively.

The full and longitudinal samples differed statistically significantly in race/ethnicity and county size, but not in mean age or type of position. The longitudinal sample had more White participants whereas the full sample had more respondents missing race/ethnicity information. The longitudinal sample had more respondents from large counties and fewer from mid-sized counties. This is likely because one large county participated in the follow-up but did not participate in the baseline survey.

### Item selection

The preliminary item pool was developed with sufficient redundancy to allow for item selection. The focus group feedback confirmed that there was indeed considerable overlap. Given the importance of parsimony in scale development in general, and the strong preference for usability and brevity in implementation science research and practice [8], we trimmed the preliminary item pool prior to conducting CFAs. We used a balanced approach to item selection informed by both expert judgment and statistical evidence. We first inspected the distribution of individual items using the follow-up survey data and found no evidence of unacceptable skewness or kurtosis. The distributional property was thus not used as a criterion to screen out items. We then examined each scale dimension and identified items that were most representative of the dimension conceptually. We also inspected inter-item correlations for each dimension to ensure that retained items had robust associations with each other (for the full correlation matrix for all preliminary items, see Additional file 1: Table S2). These considerations often converged toward the same item selection decisions. Where they did not align, we favored conceptual fit based on expert judgment.

### CFA

After item trimming, we conducted two CFAs with two forms of the scale. One was a longer form consisting of 21 items, the other a shorter form consisting of 6 items. The two forms were conceptually consistent as each sought to cover all six dimensions of PCE. The longer form was more elaborate and included multiple items for each dimension while the shorter form represented each dimension with a single item. We developed both forms anticipating they would serve different needs in research and practice. For contexts where subscale precision and reliability are desired, the longer form would be more suitable. For the general assessment of PCE, which we anticipate as the most likely scenario for PCE adoption, the shorter form would afford advantages due to its brevity at no cost to conceptual coverage. Thus, we deem the shorter form to be our final scale and will focus on the subsequent presentation of data on this scale. Analyses based on the longer form can be found in supplemental materials (Additional file 1: Supplemental Appendix B, Fig. S1, and Table S3).

**Fig. 2:**
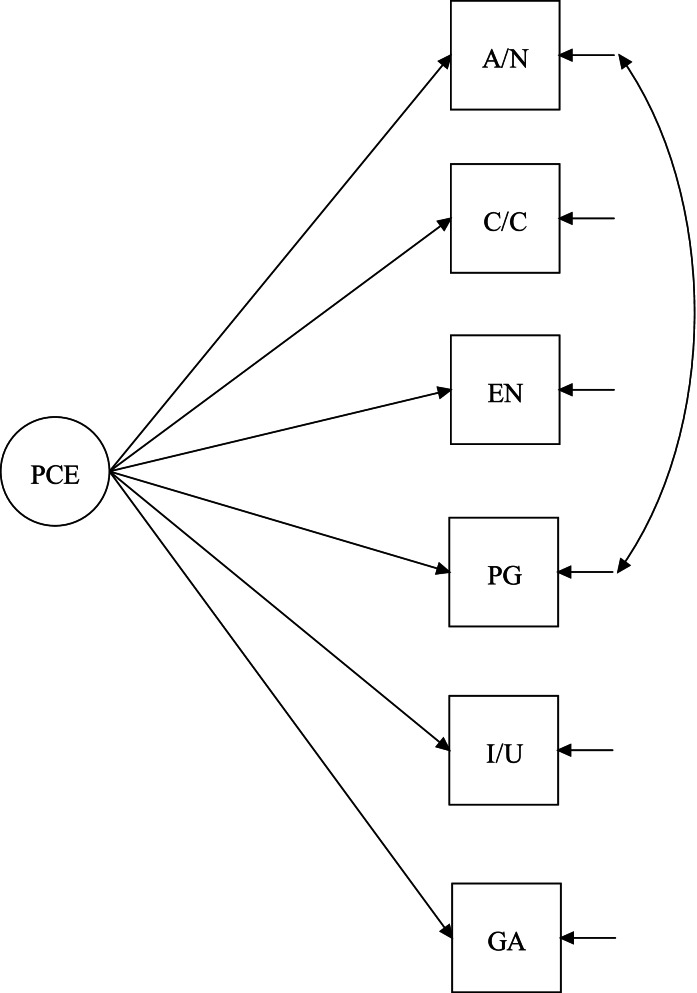
Final CFA model for 6-item scale. A/N = Attention/Novelty, C/C = Clarity/Comprehension, EN = Engagement, P/G = Perspective Gaining, I/U = Importance/ Utility; GA = General Assessment

A single factor CFA model for the final 6-item scale was fit to the data, *χ*^2^ = 51.77, df = 9, *p* < .001, CFI = .962, TLI = .937, RMSEA = .123 (90%CI = .092–.156), and SRMR = .034. While the CFI and SRMR values were adequate, the TLI and RMSEA values were not ideal, indicating room for model improvement. Inspection of modification indices suggested that the attention/novelty item and perspective gaining item shared additional covariance. Error correlations in CFAs are allowable to the extent that the additional covariance makes conceptual sense [46]. Given that the attention/novelty item and the perspective-gaining item both stressed the attainment of new information from the SUSTAIN training, it was deemed reasonable to allow for their error terms to covary. This model respecification improved fit to a satisfactory level: *χ*^2^ = 15.23, df = 8, *p* = .06, CFI = .994, TLI = .988, RMSEA = .054 (90%CI = .000–.094), and SRMR = .017. The final model is diagrammed in Fig. 2, and the factor loadings of the items are presented in Table 2.

**Table 2:** Factor loadings of the final 6-item scale

Dimension	Item	Loading
Attention/novelty	This training said something new to me.	.69
Clarity/comprehension	The information in this training was clearly presented.	.60
Engagement	I felt excited about the things I have learned from being in this training.	.89
Perspective-gaining	The content of the training opened my mind to alternative ways of thinking.	.72
Importance/utility	The ideas in this training should be implemented in my workplace.	.84
General assessment	I found myself agreeing with what this training had to say.	.86

Attention/novelty and perspective gaining correlated at .36

### Internal consistency

The 6-item scale demonstrated excellent internal consistency, Cronbach’s *α* = .898.

### Evidence of validity

Descriptives of scales used to assess the convergent, divergent, and predictive validities are shown in Table 3.

**Table 3:** Descriptives of scales used in the analysis

Scales	Baseline				Follow-up			
	*N*	Mean	SD	*α*	*N*	Mean	SD	*α*
PCE					315	4.80	1.22	.898
Acceptability [45]					284	3.44	0.92	.963
Appropriateness [45]					284	3.52	0.91	.976
Feasibility [45]					284	3.54	0.90	.957
Need to evaluate [33]					262	2.97	0.48	.751
Knowledge of EBPs	113	0.59	0.18	–	113	0.67	0.23	–
Staff use of EBPs [41, 42]	115	2.93	0.46	.841	115	2.92	0.40	.800
Agency climate [40, 43]	123	2.90	0.59	.951	123	2.91	0.55	.954
Value concordance [44]	123	3.15	0.59	.919	123	3.10	0.62	.923

#### Convergent validity

PCE was correlated with the three SUSTAIN evaluation measures. PCE was most highly correlated with the acceptability scale *r*(*N* = 272) = .819, *p* < .001, followed by the appropriateness scale *r*(*N* = 272) = .809, *p* < .001 and then the feasibility scale *r*(*N* = 272) = .754, *p* < .001. These correlations are strong evidence of convergence between PCE and the SUSTAIN-specific evaluation measures.

#### Divergent validity

There was no significant correlation between PCE and need to evaluate, *r*(*N* = 252) = − .051, *p* = .422. The lack of correlation indicates that PCE and the need to evaluate were conceptually distinct constructs.

#### Predictive validity

Evidence for PCE’s predictive validity was obtained through a series of linear regression models. Knowledge about EBPs, perceptions of staff use of EBPs, perceptions of agency learning climate, and value concordance at follow-up were each regressed on PCE, controlling for baseline values and demographics. As shown in Table 4, PCE was a significant positive predictor of staff use of EBPs (*β* = 0.230, *p* = .019), perceptions of agency climate (*β* = 0.261, *p* = .004), and value concordance (*β* = 0.209, *p* = .015) at follow-up. The association between PCE and knowledge of EBP at follow-up was positive but not significant (*β* = 0.179, *p* = .073). Taken together, these results are supportive evidence that PCE was able to predict changes in most training outcomes between baseline and follow-up.

**Table 4:** Linear regressions predicting knowledge of EBPs, perceptions of staff use of EBPs, perceptions of agency learning climate, and value concordance

Variable	Knowledge of EBPs, *β*	Use of EBPs, *β*	Perceptions of climate, *β*	Value concordance, *β*
Baseline knowledge of EBPs	0.182^†^			
Baseline use of EBPs		0.485**		
Baseline perceptions of climate			0.547**	
Baseline value concordance				0.586**
PCE	0.179^†^	0.230*	0.261*	0.209*
Age	0.052	0.174^†^	0.217*	0.217*
Non-Latinx minority	− 0.268*	− 0.027	0.080	0.063
Latinx	− 0.238*	0.013	0.057	0.130^†^
Small county	− 0.062	− 0.047	− 0.112	− 0.089
Mid-sized county	0.010	0.106	0.041	− 0.036
Officer (non-administrator)	− 0.055	0.053	0.093	0.008
*R*^2^	.234	.355	.508	.530
*F*	3.618**	6.204**	12.247**	13.365**

Coefficients are standardized regression weights

^†^*p* < .10

*p < .05

**p < .001

## Discussion

Implementation studies focus on the strategies used to facilitate organizational change or adoption of EBPs. Powell and colleagues [48, 49] identified 79 potential strategies that range from training to quality improvement practices to communication materials (e.g., pamphlets, brochures, videos). Evidence is lacking as to the effectiveness of these strategies, with a call for more studies to assess how well a strategy impacts the implementation outcomes. No instrument is readily available to measure the receptivity to each implementation strategy and whether participants have experienced a change of perspective regarding innovation or EBP. The PCE scale fills this gap and provides a tool that can be used across various implementation strategies to gauge whether the strategy resonated with participants.

The PCE scale is anchored in communication and implementation science theories and benefits from extensive groundwork in health communication contexts. Extending prior research and measurement repertoires by incorporating the needs and objectives of implementation strategies in its conceptual framework, the scale is broader in scope than previously perceived effectiveness measures. It is also highly adaptive to the wide variety of communication products and activities that may be deployed in various implementation processes. Answering the call for rigorous scale development in implementation science [8, 9], we have subjected the scale to a carefully planned, multi-phase development and validation process. The development of the scale elicited strong expertise from a diverse group of experts as well as practice-informed perspectives from staff members in a state DOC. The testing of the scale leveraged an ongoing EBP promotion effort in another state and produced data with strong ecological validity. The evidence gathered so far suggests that the scale has strong psychometric properties. Its factorial structure was clear and consistent with our conceptualization. Validity tests suggest that PCE ratings are independent from, and not biased by, individual tendencies to form judgments, and this study illustrated that the PCE was predictive of the changes in key learning outcomes targeted by the training in question. This offers strong support for PCE’s utility as both an intermediate surveillance and a diagnostic tool in the implementation process.

The predictive validity analysis in this study focused on three key training outcomes: staff use of EBPs, perceived agency climate, and value concordance. These three are implementation outcomes in the sense that they convey the extent to which the content and material would influence perceptions of others’ use of EBPs in their agency, perceptions of agency climate to support innovation, and perceptions of value concordance between an individual and the organization’s values. Each outcome confirms that PCE can predict training impacts on various dimensions of relevance. While PCE was not a statistically significant predictor of EBP knowledge, this may have more to do with the lack of sensitivity of the knowledge test. The value of the instrument is also substantiated by the convergent validity analyses which found that PCE scores were highly correlated with well-recognized implementation constructs of acceptability, appropriateness, and feasibility.

It should be noted that the question of the predictive validity of PCE is different from the one about the overall impact of the training program. As shown in Table 3, the actual outcomes of SUSTIAN did not appear to show an appreciable change between baseline and follow-up. Our analysis showed that higher PCE ratings were associated with better follow-up outcomes after controlling for baseline values. In other words, participants who rated the training as more effective displayed a greater positive change in program outcomes between baseline and follow-up. Across the entire sample, we might not have seen significant change. But within the sample, there was meaningful variation in change that was predicted by PCE. SUSTAIN only evaluated a single program, which is typically the case with training implementations in the field. Imagine that there were multiple programs being evaluated at the same time. The current evidence would suggest that programs with higher PCE ratings would lead to better outcomes. Future research should continue to validate the PCE scale using such a multiple program design, if feasible.

We prioritized brevity during the development of PCE, a preference that was strongly echoed by both focus group participants and trial users of the scale. The long form had 21 questions that included a subscale for each of the six domains (detailed in Additional file 1: Table S3). It correlated nearly perfectly with the 6-item scale and behaved similarly in validation analyses (see Additional file 1: Supplemental Appendix A). However, the shorter-form PCE better meets the needs of users, which is an important implementation outcome in its own right in terms of the willingness of participants to complete the instrument as part of the implementation activity. While the longer instrument would have provided individual scales on different domains of PCE, the shorter instrument can be more valuable in the field as an efficient tool to gauge the degree to which participants in various types of implementation strategies are receptive to the material presented.

PCE is critical for the field of implementation science, given the dearth of tools to evaluate trainings, workshops, and other communication strategies. While some tools exist to ask participants to share their experiences, these tools do not capture one important domain: whether the participant found the material compelling enough to incorporate into their ongoing routine work. Using PCE to assess the potential benefits associated with various implementation strategies can help determine which implementation strategies lead to greater changes, an unanswered question that Powell and colleagues frequently raise [48, 49]. We see PCE used primarily as a surveillance and diagnostic tool. If an implementation communication product is rated poorly on PCE (e.g., below scale midpoint) in either pretesting or actual fielding, there will be a reason to rethink whether the implementation strategy is sound. Ratings on specific scale items can give clues as to where improvement could be made. Different programs can also be compared on PCE to ascertain relative strengths and weaknesses, assuming that ratings come from the same or highly comparable samples.

This study has a few limitations. First, the predictive validity study utilized an eLearning tool (SUSTAIN) with a specific audience of probation practitioners. Future work should test the validity of PCE on other populations and dissemination materials. Second, the analyses used Weiner’s implementation outcome measures for convergent validity. While it is useful to understand acceptability, appropriateness, and feasibility, attention should also be given to other implementation outcomes such as uptake, fidelity, and cost. Finally, due to sample size restrictions, the study did not examine the differences by roles in the agencies, which may have an impact on the results. Future studies should address these limitations.

## Conclusion

PCE can be a valuable addition to the implementation toolkit in that it provides a temperature gauge on whether an individual found the communication strategy effective and useful. This is an important question since the pathway to change usually involves varied implementation strategies, but we do not understand whether the strategy clearly disseminated material that the participants found beneficial. Learning about effective implementation strategies can not only provide a better appreciation for implementation processes, but also answer the question of “how to effectively bring about change.” This question is critical to implementation science.

## Supplementary Information

**Additional file 1: Table S1.** Preliminary Items for the Perceived Communication Effectiveness (PCE) Scale. **Table S2.** Descriptive statistics of preliminary items (*N* = 315). **Table S3.** Factorial structure and loadings of the longer form PCE scale. Note. PCE22 correlated with PCE24, PCE20, and PCE25 at .522, .403, and .297, respectively. PCE24 correlated with PCE20 and PCE 25 at .409 and .439, respectively. PCE20 and PCE 25 correlated at .323. **Fig. S1.** CFA model of the longer form PCE scale. **Supplemental Appendix A.** Focus group discussion guide. **Supplemental Appendix B.** Psychometric analysis of the longer form PCE scale.

## Data Availability

The datasets used and/or analyzed during the current study are available from the corresponding author on reasonable request.
