# Supplementary material for: Perceived communication effectiveness in implementation strategies: a measurement scale
Source: Implement Sci Commun. 2022 Apr 8;3:38. doi: 10.1186/s43058-022-00284-4 (PMC8991666; doi:10.1186/s43058-022-00284-4)
Supplement: Supplementary file 1 — Additional file 1: Table S1. Preliminary Items for the Perceived Communication Effectiveness (PCE) Scale. Table S2. Descriptive statistics of preliminary items (N = 315). Table S3. Factorial structure and loadings of the longer form PCE scale. Note. PCE22 correlated with PCE24, PCE20, and PCE25 at .522, .403, and .297, respectively. PCE24 correlated with PCE20 and PCE 25 at .409 and .439, respectively. PCE20 and PCE 25 correlated at .323. Fig. S1. CFA model of the longer form PCE scale. Supplemental Appendix A. Focus group discussion guide. Supplemental Appendix B. Psychometric analysis of the longer form PCE scale. [file 43058_2022_284_MOESM1_ESM.docx]

Additional file 1

Table S1. Preliminary Items for the Perceived Effectiveness (PCE) Scale.

| Item number | Dimension | Item |
| --- | --- | --- |
| PCE_1 | Attention/novelty | The content of the training grabbed my attention. |
| PCE_10 | Attention/novelty | This training said something new to me. |
| PCE_11 | Attention/novelty | The idea in this training gives me new food for thought. |
| PCE_4 | Clarity/comprehension | The information in this training was clearly presented. |
| PCE_22 | Clarity/comprehension | The materials used in this training were confusing. |
| PCE_24 | Clarity/comprehension | The materials used in this training were difficult to follow. |
| PCE_12 | Engagement | This training made me want to learn more about this topic. |
| PCE_13 | Engagement | This training made me want to share what I learned with other people. |
| PCE_19 | Engagement | I felt excited about the things I have learned from being in this training. |
| PCE_21 | Engagement | I was more excited about the things I learned from this training than I have felt with other trainings. |
| PCE_30 | Engagement | I found it difficult to stay engaged during the training. |
| PCE_33 | Engagement | I would recommend this training to my colleagues. |
| PCE_2 | Importance/relevance | This training said something important to me. |
| PCE_3 | Importance/relevance | What I learned from this training is relevant to my work. |
| PCE_16 | Importance/relevance | This training helped me feel confident about how to best do my work. |
| PCE_17 | Importance/relevance | The ideas in this training should be implemented in my workplace. |
| PCE_18 | Importance/relevance | The ideas in this training are not something I would want to see happen in my workplace. |
| PCE_20 | Importance/relevance | The kinds of things covered in this training are not useful in practice. |
| PCE_23 | Importance/relevance | Compared to other trainings I have attended, this training helped me better understand some of the challenges we encounter at work. |
| PCE_25 | Importance/relevance | This training did not help much in terms of what I can do to improve work-related issues. |
| PCE_28 | Importance/relevance | The content of the training was mostly conventional wisdom. |
| PCE_29 | Importance/relevance | This training made me think about ways to improve what we are doing at work. |
| PCE_31 | Importance/relevance | I can use what I learned from this training immediately. |
| PCE_32 | Importance/relevance | The ideas presented in this training are similar to what we already do at work. |
| PCE_34 | Importance/relevance | I have learned concrete things from the training that can help improve procedures at work. |
| PCE_6 | Perspective-gaining | The content of the training opened my mind to alternative ways of thinking. |
| PCE_7 | Perspective-gaining | This training made me rethink some of the things about this topic. |
| PCE_14 | Perspective-gaining | This training made me reevaluate what we are currently doing at work. |
| PCE_15 | Perspective-gaining | The information in this training challenged how we handle certain issues in our work. |
| PCE_26 | Perspective-gaining | The training helped me to think outside of the box. |
| PCE_8 | General agreement/quality | I found myself agreeing with what this training had to say. |
| PCE_9 | General agreement/quality | Overall, this training was convincing. |
| PCE_5 | General agreement/quality | The information in this training was believable. |
| PCE_27 | General agreement/quality | I am not sure I can trust the information presented in the training. |

Table S2. Descriptive statistics of preliminary items (N = 315)

|  | PCE1 | PCE2 | PCE3 | PCE4 | PCE5 | PCE6 | PCE7 | PCE8 | PCE9 | PCE10 | PCE11 | PCE12 | PCE13 | PCE14 | PCE15 | PCE16 | PCE17 |
| --- | --- | --- | --- | --- | --- | --- | --- | --- | --- | --- | --- | --- | --- | --- | --- | --- | --- |
| PCE2 | 0.742 |  |  |  |  |  |  |  |  |  |  |  |  |  |  |  |  |
| PCE3 | 0.645 | 0.694 |  |  |  |  |  |  |  |  |  |  |  |  |  |  |  |
| PCE4 | 0.574 | 0.478 | 0.498 |  |  |  |  |  |  |  |  |  |  |  |  |  |  |
| PCE5 | 0.689 | 0.751 | 0.728 | 0.542 |  |  |  |  |  |  |  |  |  |  |  |  |  |
| PCE6 | 0.69 | 0.722 | 0.611 | 0.409 | 0.605 |  |  |  |  |  |  |  |  |  |  |  |  |
| PCE7 | 0.664 | 0.713 | 0.553 | 0.351 | 0.623 | 0.746 |  |  |  |  |  |  |  |  |  |  |  |
| PCE8 | 0.715 | 0.74 | 0.75 | 0.552 | 0.823 | 0.592 | 0.61 |  |  |  |  |  |  |  |  |  |  |
| PCE9 | 0.79 | 0.78 | 0.696 | 0.568 | 0.821 | 0.674 | 0.703 | 0.811 |  |  |  |  |  |  |  |  |  |
| PCE10 | 0.636 | 0.668 | 0.527 | 0.395 | 0.604 | 0.677 | 0.688 | 0.562 | 0.659 |  |  |  |  |  |  |  |  |
| PCE11 | 0.757 | 0.765 | 0.677 | 0.458 | 0.664 | 0.786 | 0.732 | 0.667 | 0.741 | 0.702 |  |  |  |  |  |  |  |
| PCE12 | 0.797 | 0.782 | 0.647 | 0.467 | 0.684 | 0.752 | 0.703 | 0.708 | 0.775 | 0.678 | 0.778 |  |  |  |  |  |  |
| PCE13 | 0.815 | 0.779 | 0.67 | 0.508 | 0.71 | 0.693 | 0.676 | 0.756 | 0.818 | 0.656 | 0.741 | 0.83 |  |  |  |  |  |
| PCE14 | 0.538 | 0.547 | 0.464 | 0.322 | 0.468 | 0.634 | 0.591 | 0.455 | 0.522 | 0.546 | 0.612 | 0.617 | 0.559 |  |  |  |  |
| PCE15 | 0.535 | 0.605 | 0.476 | 0.241 | 0.483 | 0.597 | 0.631 | 0.454 | 0.531 | 0.559 | 0.674 | 0.58 | 0.521 | 0.573 |  |  |  |
| PCE16 | 0.735 | 0.727 | 0.646 | 0.489 | 0.689 | 0.684 | 0.672 | 0.718 | 0.767 | 0.648 | 0.718 | 0.718 | 0.756 | 0.539 | 0.55 |  |  |
| PCE17 | 0.708 | 0.755 | 0.712 | 0.498 | 0.742 | 0.6 | 0.619 | 0.742 | 0.765 | 0.55 | 0.666 | 0.731 | 0.751 | 0.543 | 0.528 | 0.72 |  |
| PCE18 | -0.443 | -0.447 | -0.452 | -0.317 | -0.468 | -0.347 | -0.322 | -0.516 | -0.467 | -0.32 | -0.412 | -0.437 | -0.477 | -0.187 | -0.252 | -0.477 | -0.506 |
| PCE19 | 0.81 | 0.776 | 0.666 | 0.531 | 0.716 | 0.677 | 0.67 | 0.755 | 0.835 | 0.65 | 0.74 | 0.789 | 0.837 | 0.58 | 0.533 | 0.765 | 0.737 |
| PCE20 | -0.422 | -0.435 | -0.45 | -0.336 | -0.452 | -0.332 | -0.295 | -0.507 | -0.488 | -0.264 | -0.387 | -0.424 | -0.454 | -0.203 | -0.203 | -0.389 | -0.482 |
| PCE21 | 0.749 | 0.678 | 0.541 | 0.494 | 0.602 | 0.622 | 0.593 | 0.622 | 0.7 | 0.547 | 0.625 | 0.708 | 0.738 | 0.553 | 0.476 | 0.67 | 0.642 |
| PCE22 | -0.387 | -0.336 | -0.31 | -0.524 | -0.401 | -0.241 | -0.211 | -0.38 | -0.414 | -0.241 | -0.231 | -0.312 | -0.399 | -0.198 | -0.073 | -0.345 | -0.357 |
| PCE23 | 0.725 | 0.734 | 0.583 | 0.492 | 0.64 | 0.7 | 0.677 | 0.645 | 0.742 | 0.664 | 0.711 | 0.745 | 0.727 | 0.616 | 0.566 | 0.737 | 0.672 |
| PCE24 | -0.372 | -0.243 | -0.265 | -0.517 | -0.338 | -0.188 | -0.172 | -0.351 | -0.402 | -0.205 | -0.237 | -0.268 | -0.347 | -0.16 | -0.022 | -0.281 | -0.269 |
| PCE25 | -0.512 | -0.506 | -0.403 | -0.34 | -0.459 | -0.433 | -0.412 | -0.446 | -0.502 | -0.379 | -0.451 | -0.509 | -0.526 | -0.316 | -0.285 | -0.452 | -0.468 |
| PCE26 | 0.744 | 0.721 | 0.633 | 0.457 | 0.653 | 0.777 | 0.712 | 0.645 | 0.756 | 0.702 | 0.823 | 0.787 | 0.771 | 0.613 | 0.649 | 0.729 | 0.673 |
| PCE27 | -0.463 | -0.479 | -0.497 | -0.393 | -0.612 | -0.399 | -0.399 | -0.612 | -0.552 | -0.374 | -0.489 | -0.487 | -0.514 | -0.281 | -0.29 | -0.443 | -0.503 |
| PCE28 | 0.15 | 0.103 | 0.14 | 0.147 | 0.167 | 0.095 | 0.053 | 0.21 | 0.138 | 0.047 | 0.072 | 0.098 | 0.105 | 0.05 | 0.079 | 0.184 | 0.122 |
| PCE29 | 0.672 | 0.683 | 0.658 | 0.433 | 0.643 | 0.729 | 0.726 | 0.67 | 0.739 | 0.636 | 0.752 | 0.736 | 0.714 | 0.571 | 0.541 | 0.682 | 0.683 |
| PCE30 | -0.607 | -0.486 | -0.392 | -0.427 | -0.462 | -0.453 | -0.416 | -0.476 | -0.547 | -0.435 | -0.487 | -0.526 | -0.559 | -0.302 | -0.313 | -0.494 | -0.441 |
| PCE31 | 0.657 | 0.722 | 0.749 | 0.516 | 0.735 | 0.58 | 0.591 | 0.745 | 0.722 | 0.542 | 0.704 | 0.629 | 0.669 | 0.452 | 0.437 | 0.68 | 0.671 |
| PCE32 | 0.209 | 0.233 | 0.428 | 0.274 | 0.322 | 0.182 | 0.137 | 0.325 | 0.266 | 0.082 | 0.214 | 0.134 | 0.191 | 0.024 | 0.061 | 0.223 | 0.277 |
| PCE33 | 0.8 | 0.781 | 0.669 | 0.602 | 0.773 | 0.663 | 0.652 | 0.78 | 0.84 | 0.624 | 0.735 | 0.768 | 0.829 | 0.514 | 0.51 | 0.755 | 0.789 |
| PCE34 | 0.786 | 0.795 | 0.72 | 0.529 | 0.769 | 0.717 | 0.703 | 0.779 | 0.804 | 0.667 | 0.765 | 0.751 | 0.784 | 0.558 | 0.569 | 0.767 | 0.722 |
| Mean | 4.44 | 4.83 | 5.66 | 5.11 | 5.18 | 4.78 | 4.66 | 5.13 | 4.85 | 4.48 | 4.96 | 4.47 | 4.41 | 4.23 | 4.57 | 4.62 | 4.98 |
| SD | 1.612 | 1.526 | 1.206 | 1.434 | 1.418 | 1.458 | 1.503 | 1.411 | 1.534 | 1.601 | 1.407 | 1.599 | 1.591 | 1.56 | 1.587 | 1.585 | 1.511 |
| Skewness | -0.531 | -0.79 | -1.288 | -1.102 | -1.213 | -0.724 | -0.688 | -0.987 | -0.765 | -0.649 | -0.954 | -0.506 | -0.459 | -0.269 | -0.556 | -0.704 | -0.77 |
| Kurtosis | -0.494 | 0.077 | 2.146 | 0.898 | 1.288 | 0.042 | -0.08 | 0.719 | -0.032 | -0.353 | 0.519 | -0.379 | -0.512 | -0.69 | -0.512 | -0.194 | 0.009 |

Table S2. (continued)

|  | PCE18 | PCE19 | PCE20 | PCE21 | PCE22 | PCE23 | PCE24 | PCE25 | PCE26 | PCE27 | PCE28 | PCE29 | PCE30 | PCE31 | PCE32 | PCE33 | PCE34 |
| --- | --- | --- | --- | --- | --- | --- | --- | --- | --- | --- | --- | --- | --- | --- | --- | --- | --- |
| PCE19 | -0.492 |  |  |  |  |  |  |  |  |  |  |  |  |  |  |  |  |
| PCE20 | 0.555 | -0.503 |  |  |  |  |  |  |  |  |  |  |  |  |  |  |  |
| PCE21 | -0.396 | 0.752 | -0.31 |  |  |  |  |  |  |  |  |  |  |  |  |  |  |
| PCE22 | 0.375 | -0.411 | 0.505 | -0.35 |  |  |  |  |  |  |  |  |  |  |  |  |  |
| PCE23 | -0.375 | 0.771 | -0.395 | 0.743 | -0.362 |  |  |  |  |  |  |  |  |  |  |  |  |
| PCE24 | 0.329 | -0.368 | 0.484 | -0.268 | 0.69 | -0.289 |  |  |  |  |  |  |  |  |  |  |  |
| PCE25 | 0.485 | -0.529 | 0.522 | -0.442 | 0.436 | -0.444 | 0.501 |  |  |  |  |  |  |  |  |  |  |
| PCE26 | -0.4 | 0.768 | -0.377 | 0.659 | -0.338 | 0.736 | -0.275 | -0.47 |  |  |  |  |  |  |  |  |  |
| PCE27 | 0.584 | -0.535 | 0.602 | -0.376 | 0.439 | -0.414 | 0.434 | 0.543 | -0.439 |  |  |  |  |  |  |  |  |
| PCE28 | 0.103 | 0.098 | 0.117 | 0.152 | 0.093 | 0.104 | 0.109 | 0.162 | 0.078 | 0.022 |  |  |  |  |  |  |  |
| PCE29 | -0.38 | 0.738 | -0.398 | 0.608 | -0.287 | 0.66 | -0.279 | -0.457 | 0.749 | -0.479 | 0.122 |  |  |  |  |  |  |
| PCE30 | 0.5 | -0.568 | 0.58 | -0.477 | 0.583 | -0.521 | 0.593 | 0.568 | -0.516 | 0.531 | 0.121 | -0.434 |  |  |  |  |  |
| PCE31 | -0.482 | 0.686 | -0.526 | 0.552 | -0.346 | 0.591 | -0.314 | -0.416 | 0.638 | -0.579 | 0.107 | 0.65 | -0.462 |  |  |  |  |
| PCE32 | -0.129 | 0.173 | -0.157 | 0.145 | -0.072 | 0.133 | -0.061 | 0.012 | 0.196 | -0.148 | 0.287 | 0.165 | -0.039 | 0.386 |  |  |  |
| PCE33 | -0.523 | 0.79 | -0.513 | 0.723 | -0.468 | 0.736 | -0.411 | -0.507 | 0.768 | -0.543 | 0.12 | 0.672 | -0.583 | 0.721 | 0.223 |  |  |
| PCE34 | -0.444 | 0.79 | -0.425 | 0.699 | -0.389 | 0.734 | -0.334 | -0.527 | 0.779 | -0.518 | 0.155 | 0.738 | -0.518 | 0.72 | 0.234 | 0.78 |  |
| Mean | 2.91 | 4.31 | 2.96 | 3.81 | 3.46 | 4.44 | 3.42 | 3.33 | 4.69 | 2.6 | 4.31 | 5.08 | 4.01 | 5.26 | 5.54 | 4.8 | 4.89 |
| Std. Deviation | 1.606 | 1.538 | 1.623 | 1.591 | 1.717 | 1.59 | 1.718 | 1.645 | 1.518 | 1.471 | 1.478 | 1.385 | 1.798 | 1.453 | 1.115 | 1.72 | 1.478 |
| Skewness | 0.721 | -0.427 | 0.769 | 0.006 | 0.362 | -0.573 | 0.375 | 0.479 | -0.588 | 1.04 | -0.291 | -0.919 | 0.022 | -1.263 | -1.147 | -0.653 | -0.801 |
| Kurtosis | -0.339 | -0.374 | -0.334 | -0.619 | -0.989 | -0.443 | -1 | -0.768 | -0.32 | 0.627 | -0.482 | 0.529 | -1.182 | 1.418 | 2.275 | -0.528 | 0.171 |

Table S3. Factorial structure and loadings of the longer form PCE scale.

| First Level |  |  |  |
| --- | --- | --- | --- |
| Dimension | Item | Loading | Alpha |
| Attention/Novelty | PCE1 | .862 |  |
| Attention/Novelty | PCE10 | .786 |  |
| Attention/Novelty | PCE11 | .881 |  |
| Clarity/Comprehension | PCE4 | .868 |  |
| Clarity/Comprehension | PCE22 | -.602 |  |
| Clarity/Comprehension | PCE24 | -.579 |  |
| Engagement | PCE12 | .882 |  |
| Engagement | PCE13 | .910 |  |
| Engagement | PCE19 | .899 |  |
| Engagement | PCE33 | .904 |  |
| Perspective Gaining | PCE6 | .862 |  |
| Perspective Gaining | PCE7 | .821 |  |
| Perspective Gaining | PCE26 | .901 |  |
| Importance/Utility | PCE2 | .881 |  |
| Importance/Utility | PCE16 | .845 |  |
| Importance/Utility | PCE17 | .848 |  |
| Importance/Utility | PCE20 | -.521 |  |
| Importance/Utility | PCE25 | -.574 |  |
| General Assessment | PCE5 | .878 |  |
| General Assessment | PCE8 | .882 |  |
| General Assessment | PCE9 | .940 |  |
| Second Level |  |  |  |
| PCE | Attention/Novelty | .971 | .871 |
| PCE | Clarity/Comprehension | .681 | .787 |
| PCE | Engagement | 1.000 | .943 |
| PCE | Perspective Gaining | .917 | .897 |
| PCE | Importance/Utility | .991 | .847 |
| PCE | General Assessment | .953 | .930 |

Note. PCE22 correlated with PCE24, PCE20, and PCE25 at .522, .403, and .297, respectively. PCE24 correlated with PCE20 and PCE 25 at .409 and .439, respectively. PCE20 and PCE 25 correlated at .323.

Figure S1. CFA model of the longer form PCE scale

**
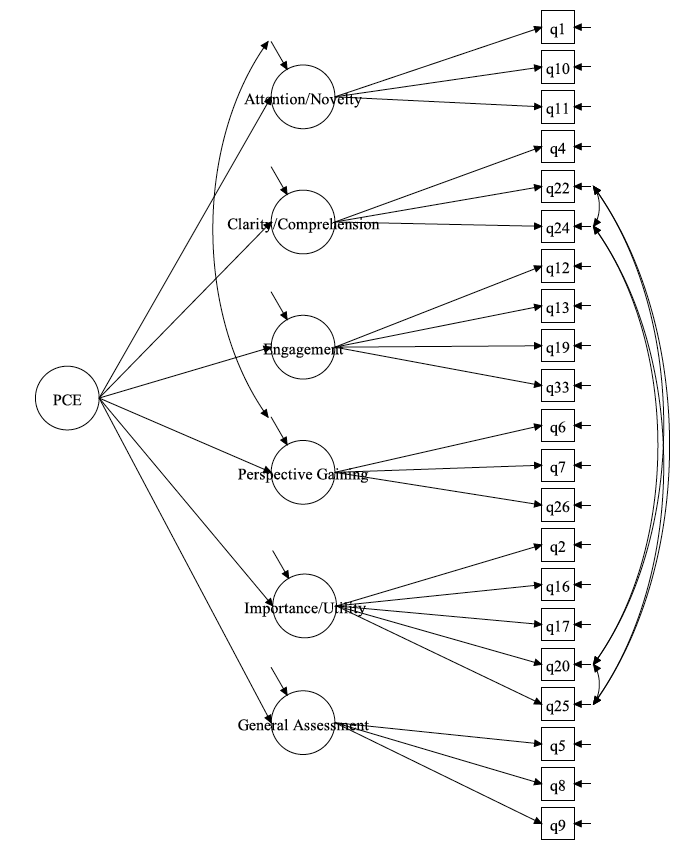
**

Supplemental Appendix A. Focus group discussion guide

Hello everyone, our names are [introduce focus group moderators]. We wanted to thank you for taking the time to participate in our focus group research. We are researchers from George Mason University and are part of a team being led by Dr. XXX and Dr. XXX under an initiative called Justice Community Opioid Innovation Network, also known as JCOIN. We are conducting this focus group to assess the clarity, appropriateness, and ease of understanding of two groups of questions that are designed to determine the perceived effectiveness and extent of learning achieved in the Academy training you received earlier.

Our discussion today should take about 1 hour, depending on how you elaborate in your answers. Since this is a group discussion, you are encouraged to talk to each other in your responses. Please note that there are no right or wrong answers in this discussion. We are testing some survey questions, not you. We want to know your opinions and what you think. We do not work for the training provider or XXX DOC, so please don’t hold back from giving us your honest opinions.

The discussion we will have today is confidential. You are not obligated to answer any questions you do not want to, and you may withdraw your participation from the research at any time. If you have any follow-up questions or concerns, we can send you the consent form that has the contact information for us, the principle investigator on the project, as well as XXX University’s IRB Office.

Do you have any questions before we begin?

Now we would like to begin recording our discussion, and once we begin recording we would like to ask for each of your permission again so that we have it for the record.

[Turn on recorder.]

Do I have all of your permission to record our discussion today? [Wait for agreement from each participant.] Great, thank you all!

Please feel free to use the chat room to share thoughts as well but you don’t have to.

**Warm-Up** (~5 minutes)

I hope everybody is doing well with the COVID-19 outbreak still going on. Talking about COVID-19,

How has it affected your work in Iowa?

Which of the changes have staff found to be acceptable? Do you think they will lead to any long-term changes in your organization?

**In Your Own Words** (~10 minutes)

You have all participated in the implementation leadership academy.

How would you evaluate the training?

What did you like about it?

Were there things you dislike?

If you have to be critical, what would you say about the experience?

**Perceived Effectiveness Scale** (~35 minutes)

We have sent you a questionnaire. Part 1 asks about your perceptions of the effectiveness of the training. Some of the questions were included in the online survey you may have recently completed. But there are also some revised and new questions. We will share our screen with you that shows the questions we will be discussing. If it is hard for you to see the questions on your screen, it may be helpful if you have the questionnaire file we sent you open on your computer or have a hard copy of the questionnaire in front of you.

Now let’s review these questions. We will look at them in sections. We will first look at questions 1 through 11, then questions 12 through 22, and finally questions 23-34. [Rotate order for different groups/interviews.]

[For each group]

Please take a moment to review the questions.

**What are your thoughts about these questions/statements?**

CONTENT PROBE:

Do these questions challenge you to assess the value of this training?

Are any of the questions difficult for you to assess?

Can you identify key ideas that would help others assess the value attributed to the training?

Which of the questions seem poorly-suited to the training you received?

Why do they seem inappropriate?

PRESENTATION PROBE:

Are any of the items confusing to you?

Why are they confusing?

Anything seem unclear to you?

Why does it seem unclear?

Does any question seem to be worded strangely?

How might you re-word the question to be clearer and less confusing?

TARGETED PROBE [if the items did not come up in open discussion]:

What about this particular item?

What does it mean to you?

What did you come up with your answer to this question?

[After all three groups are discussed]

FINAL PROBE: Looking at the questionnaire as a whole…(~5 minutes)

Which items would you consider removing from this list?

Why would you choose to remove those items?

How might you re-word any of the questions to be more appropriate?

Are there questions you might want to add to this list? Why?

**Wrap-Up**

Thank you for participating in today’s focus group. You have been most helpful!

Supplemental Appendix B. Psychometric analysis of the longer form PCE scale

The longer form scale had 3 items each for attention/novelty, clarity/comprehension, perspective gaining, general agreement, and 4 items each for engagement and utility/importance. A two-level CFA model was specified and fit to the data. Initial estimation returned less than ideal fit indices: χ2 = 722.05, df = 183, p < .001, CFI = .920, TLI = .909, RMSEA = .097 (90%CI = .089 - .104), SRMR = .071. Inspection of model results and modification indices suggested two potential changes that could improve model fit. The first was to allow the negatively worded items in the scale to correlate. This is an often-observed source of covariation among items and reasonable cause for model respecification [35]. The other was to allow the first-order factors of attention/novelty and perspective gaining to correlate. As explained in the presentation of the final 6-item scale, this modification was deemed substantively meaningful and permissible. After making these two changes, the model produced much better fit to the data: χ2 = 473.72, df = 176, p < .001, CFI = .956, TLI = .948, RMSEA = .073 (90%CI = .065 - .081), SRMR = .038. While RMSEA was still slightly above the ideal range, all other fit indices indicated adequate fit. All considered, the model fit was acceptable. The model is presented in Supplemental Figure 1 and factor loadings are presented in Supplemental Table 3.

The 21-item scale correlated with the 6-item scale at *r* = .975, p < .001, suggesting strong equivalence between them. Validation analysis using this longer form produced nearly identical results as those obtained for the 6-item scale (details not reported).
